# Blood cells morphology change with COVID-19

**DOI:** 10.11604/pamj.supp.2020.35.2.23557

**Published:** 2020-05-21

**Authors:** Maryame Ahnach, Fadwa Ousti

**Affiliations:** 1 Department of Hematology, Cheikh Khalifa International University Hospital, Mohammed VI University of Health Sciences Casablanca, Morocco; 2 National Reference Laboratory, Mohammed VI University of Health Sciences Casablanca, Morocco

**Keywords:** COVID-19, blood cell, morphology

## Image in medicine

Hematologic abnormalities are reported in Coronavirus disease (COVID-19) infection, all blood cells can be affected, mainly leukocyte and platelet cells. blood smears are not systematically requested, but given the quantitative abnormalities, a qualitative evaluation is therefore useful to analyze cytological changes during COVID-19. A 75-years old women with history of ischemic cardiopathy, presented to the emergency department with cough and dyspnea. She had a contact exposure with her son, infected by the new coronavirus (COVID-19). Chest X-ray and CT scan found a typical lung lesions classified CORADS 4. The viral detection of naso-pharyngeal swabs by RT-PCR confirmed that the patient was positive for COVID-19. Her initial complete blood count showed a sever neutropenia (730/ul), lymphopenia (900/ul) with normal hemoglobin and platelet count. The peripheral blood smear revealed many morphological abnormalities, neutrophil granulocyte with marked hypogranular cytoplasm and hyposegmented nucleus, eosinophil containing multiple vacuoles and circulating of rare large lymphocytes. The clinical condition of patient was stable at admission, classified in moderate severity, she was treated with chloroquine and azithromycin during 10 days, but two control PCR tests were still positive. The patient was transferred to intensive unit after respiratory worsening, her biological analysis confirmed the persistence of lymphopenia and neutropenia.

**Figure 1:**
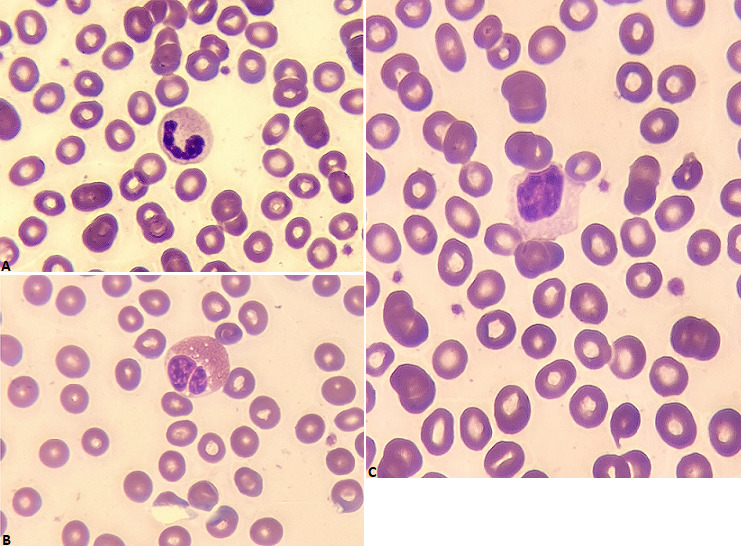
A) neutrophil granulocyte with marked hypogranular cytoplasm and hyposegmented nucleus (may grunwald giemsa MGG); B) eosinophil containing multiple vacuoles; C) circulating of rare large lymphocytes

